# A Functional Genomics Approach Identifies Candidate Effectors from the Aphid Species *Myzus persicae* (Green Peach Aphid)

**DOI:** 10.1371/journal.pgen.1001216

**Published:** 2010-11-18

**Authors:** Jorunn I. B. Bos, David Prince, Marco Pitino, Massimo E. Maffei, Joe Win, Saskia A. Hogenhout

**Affiliations:** 1 Department of Disease and Stress Biology, The John Innes Centre, Norwich, United Kingdom; 2 Plant Physiology Unit, Department of Plant Biology and Centre of Excellence CEBIOVEM, University of Turin, Turin, Italy; 3 The Sainsbury Laboratory, Norwich, United Kingdom; The University of North Carolina at Chapel Hill, United States of America

## Abstract

Aphids are amongst the most devastating sap-feeding insects of plants. Like most plant parasites, aphids require intimate associations with their host plants to gain access to nutrients. Aphid feeding induces responses such as clogging of phloem sieve elements and callose formation, which are suppressed by unknown molecules, probably proteins, in aphid saliva. Therefore, it is likely that aphids, like plant pathogens, deliver proteins (effectors) inside their hosts to modulate host cell processes, suppress plant defenses, and promote infestation. We exploited publicly available aphid salivary gland expressed sequence tags (ESTs) to apply a functional genomics approach for identification of candidate effectors from *Myzus persicae* (green peach aphid), based on common features of plant pathogen effectors. A total of 48 effector candidates were identified, cloned, and subjected to transient overexpression in *Nicotiana benthamiana* to assay for elicitation of a phenotype, suppression of the Pathogen-Associated Molecular Pattern (PAMP)–mediated oxidative burst, and effects on aphid reproductive performance. We identified one candidate effector, Mp10, which specifically induced chlorosis and local cell death in *N. benthamiana* and conferred avirulence to recombinant *Potato virus X* (PVX) expressing Mp10, PVX-Mp10, in *N. tabacum*, indicating that this protein may trigger plant defenses. The ubiquitin-ligase associated protein SGT1 was required for the Mp10-mediated chlorosis response in *N. benthamiana*. Mp10 also suppressed the oxidative burst induced by flg22, but not by chitin. Aphid fecundity assays revealed that *in planta* overexpression of Mp10 and Mp42 reduced aphid fecundity, whereas another effector candidate, MpC002, enhanced aphid fecundity. Thus, these results suggest that, although Mp10 suppresses flg22-triggered immunity, it triggers a defense response, resulting in an overall decrease in aphid performance in the fecundity assays. Overall, we identified aphid salivary proteins that share features with plant pathogen effectors and therefore may function as aphid effectors by perturbing host cellular processes.

## Author Summary

Aphids are insects that can induce feeding damage, achieve high population densities, and most importantly, transmit economically important plant diseases worldwide. To develop durable approaches to control aphids, it is critical to understand how aphids interact with plants at the molecular level. Aphid feeding induces plant defenses, which can be suppressed by aphid saliva. Thus, aphids can alter plant cellular processes to promote infestation of plants. Suppression of plant defenses is common in plant pathogens and involves secretion of effector proteins that modulate host cell processes. Evidence suggests that aphids, like plant pathogens, deliver effectors inside their host cells to promote infestation. However, the identity of these effectors and their functions remain elusive. Here, we report a novel approach based on a combination of bioinformatics and functional assays to identify candidate effectors from the aphid species *Myzus persicae*. Using this approach, we identified three candidate effectors that affect plant defense responses and/or aphid reproductive performance. Further characterization of these candidates promises to reveal new insights into the plant cellular processes targeted by aphids.

## Introduction

Like most plant parasites, aphids require intimate associations with their host plants to gain access to nutrients. Aphids predominantly feed from the plant phloem sieve elements, and use their stylets to navigate between the cells of different layers of leaf tissue during which plant defenses may be triggered. Indeed, aphid feeding induces responses such as clogging of phloem sieve elements and callose formation, which are suppressed by the aphid in successful interactions with plant hosts [1]. In addition, some aphid species can alter host plant phenotypes, by for example inducing the formation of galls or causing leaf curling [2] indicating that there is an active interplay between host and aphid at the molecular level. During probing and feeding, aphids secrete two types of saliva: gelling saliva, which is thought to protect stylets during penetration, and watery saliva, which is secreted into various plant host cell types and the phloem [3]. The secretion of aphid saliva directly into the host-stylet interface [4], suggests that molecules present in the saliva may perturb plant cellular processes while aphids progress through different feeding stages. Interestingly, the knock-down of the *C002* salivary gene in *Acyrthosiphon pisum* (pea aphid) negatively impacts survival rates of this aphid on plant hosts [5], [6]. Furthermore, proteomics studies based on artificial aphid diets showed the presence of secreted proteins, including C002, in aphid saliva indicating that these proteins are delivered inside the host plant during feeding [7], [8]. However, whether and how these aphid salivary proteins function in the plant host remains elusive.

Suppression of host defenses and altering host plant phenotypes is common in plant-pathogen interactions and involves secretion of molecules (effectors) that modulate host cell processes [9], [10]. Therefore it is likely that aphids, similar to plant pathogens, deliver effectors inside their hosts to manipulate host cell process enabling successful infestation of plants [9]. Effector-mediated suppression of plant defenses, such as Pathogen-Associated Molecular Pattern (PAMP)-triggered immunity (PTI), generally involves the targeting of a plant virulence target, or operative target [11]. However, plant pathogen effectors that are deployed to suppress host defenses are recognized by plant disease resistance (R) proteins in particular host genotypes, resulting in effector-triggered immunity (ETI) [12]. Interestingly, the R proteins that recognize plant pathogens and those that confer resistance to aphids, such as *Mi-1.2* and *Vat*, share a similar structure, and contain a nucleotide binding site (NBS) domain and leucine rich repeat (LRR) regions [13]–[15]. The *Mi-1.2* resistance gene confers resistance in tomato to certain clones of *Macrosiphum euphorbiae* (potato aphid), two whitefly biotypes, a psyllid, and three nematode species [16]–[19], indicating that there is significant overlap in plant pathogen and aphid recognition in plants. In addition, aphid resistance conferred by several resistance genes was shown to be race-specific [16], [20]. This suggests that depending on their genotype, certain aphid clones may be able to avoid and/or suppress plant defenses and fits with the gene-for-gene model in plant-pathogen interactions [21]. Therefore, it is likely that not only plant pathogens, but also aphids, secrete effectors that in addition to targeting host cell processes may trigger ETI depending on the host genotype.

Plant pathogen effectors generally share the common feature of modulating host cell processes [22]. Various assays have been developed to identify the functions of effectors from bacterial and eukaryotic filamentous plant pathogens [22]–[24]. One important and common function of plant pathogen effectors is the suppression of PTI. This activity is especially common among type III secretion system (T3SS) effectors. For example, the large majority of *Pseudomonas syringae* DC3000 effectors can suppress PTI responses, including the oxidative burst [25]. However, effectors from eukaryotic filamentous plant pathogens can also suppress PTI, as demonstrated for the AVR3a effector from *Phytophthora infestans*, which suppresses cell death induced by the PAMP-like elicitor INF1 [26], [27]. Another activity of plant pathogen effectors is the induction of phenotypes in plants. For example, several effectors, including CRN2 and INF1, from the oomycete plant pathogen *P. infestans* induce cell death upon overexpression *in planta* [28], [29], whereas other effectors, like AvrB from *P. syringae* DC3000 induce chlorosis [30]. Also, overexpression of effector proteins from plant pathogenic nematodes in host plants can affect plant phenotypes, as shown for the *Heterodera glycines* CLE protein Hg-SYV46 that alters host cell differentiation [31]. As effectors exhibit functions important for pathogenicity, their deletion can have detrimental effects on pathogen virulence. However, due to redundancy, the knock-down or deletion of single effectors does not always impact virulence. On the other hand, overexpression of plant pathogen effectors can enhance pathogen virulence, as shown for active AvrPtoB, which enhances virulence to *P. syringae* DC3000 in Arabidopsis [32], and for the *H. schachtii* effector 10A06 that, in addition to altering host plant morphology, increases nematode susceptibility in *Arabidopsis* [33].

We exploited publicly available aphid salivary gland sequences to develop a functional genomics approach for the identification of candidate aphid effector proteins from the aphid species *Myzus persicae* (green peach aphid) based on common features of plant pathogen effectors. Data mining of salivary gland expressed sequences tags (ESTs) identified 46 *M. persicae* predicted secreted proteins. Functional analyses showed that one of these proteins, Mp10, induced chlorosis and weak cell death in *Nicotiana benthamiana*, and suppressed the oxidative burst induced by the bacterial PAMP flg22. In addition, we developed a medium-throughput assay, based on transient overexpression in *N. benthamiana*, that allows screening for effects of aphid candidate effectors on aphid performance. Using this screen, we identified two candidate effectors, Mp10 and Mp42, that reduced aphid performance and one effector candidate, MpC002, that enhanced aphid performance. In summary, we found aphid secreted salivary proteins that share features with plant pathogen effectors and therefore may function as aphid effectors by perturbing host cellular processes.

## Results

### Description of functional genomics screen

**Figure 1:**
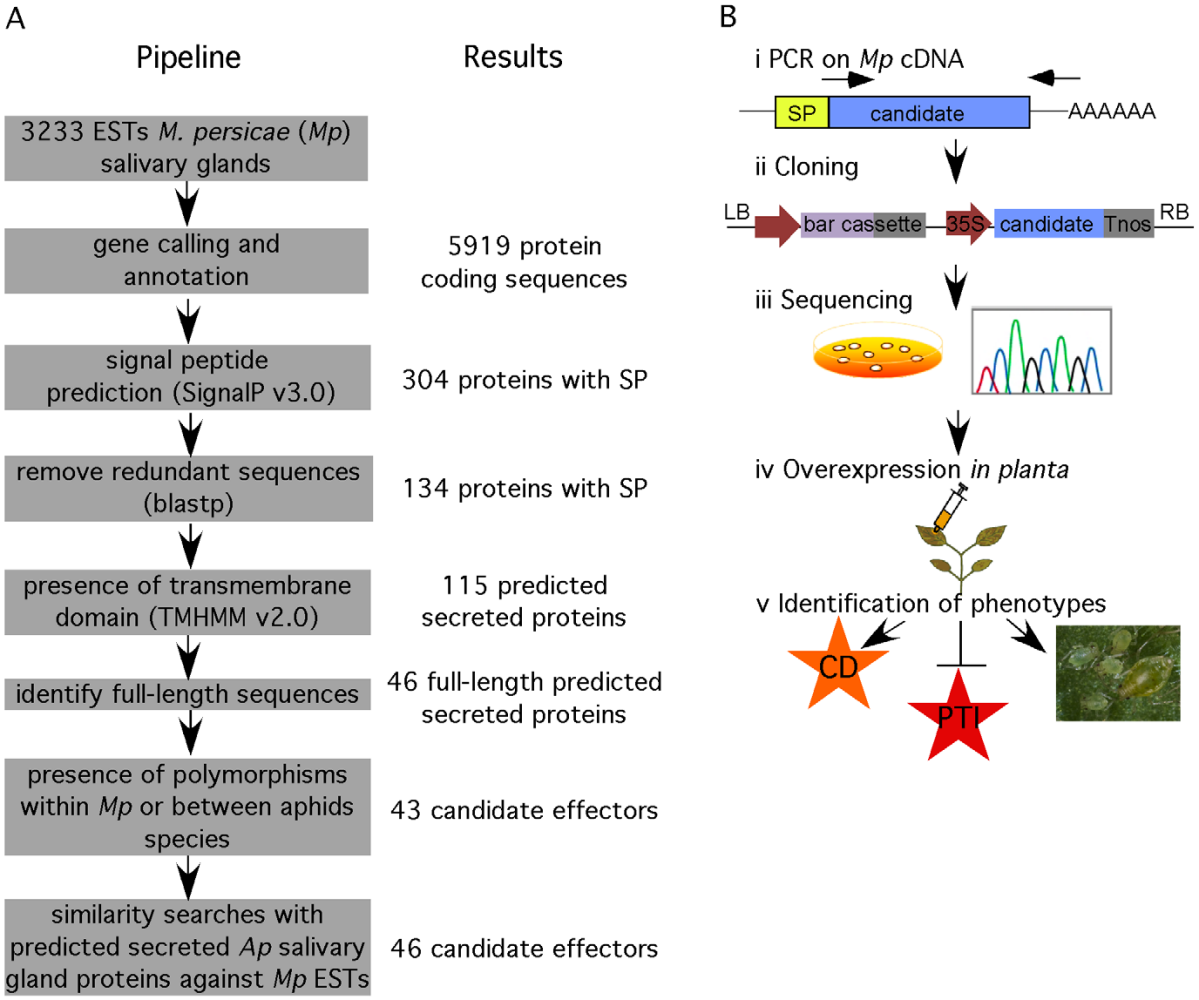
Overview of functional genomics pipeline to identify candidate effectors from *M. persicae*. (A) Bioinformatics pipeline for data mining of *M. persicae* salivary gland expressed sequence tags (ESTs). (B) Cloning and functional analyses of candidates to identify effector activities. i) PCR amplification was performed on *M. persicae* cDNA. ii) Amplicons were cloned in the pCB302-3 vector under control of a 35S promoter and constructs were transformed into *Agrobacterium tumefaciens*. iii) Multiple clones were sequenced to identify polymorphic candidates. Clones were stored and cultured for subsequent functional assays. iv) Candidate effectors were overexpressed in *Nicotiana benthamiana* by agroinfiltration to determine whether they induce a phenotype *in planta*, such as cell death, suppress basal plant defences, PAMP-triggered immunity (PTI), and affect aphid performance.

We developed a functional genomics approach to identify candidate effectors from *M. persicae* using 3233 publicly available aphid salivary gland ESTs [34]. We hypothesized that aphid effectors are most likely secreted proteins that are delivered into the saliva through the classical eukaryotic endoplasmic reticulum (ER)-Golgi pathway of the salivary glands. A feature of proteins secreted through this pathway is the presence of an N-terminal signal peptide. Therefore, we used the SignalP v3.0 program [35] to predict the presence of signal peptides in the amino acid sequences encoded by the open reading frames (ORFs) found in salivary gland ESTs. Out of 5919 amino acid sequences corresponding to predicted ORFs, we identified 134 nonredundant sequences with signal peptide (Figure 1A). Out of these 134 proteins, 19 were predicted to contain a transmembrane domain in addition to the signal peptide, and are therefore likely to remain in the salivary gland membrane upon secretion. Hence, 115 predicted secreted proteins remained. In order to investigate the *M. persicae* candidate effector protein in functional assays, we started with the cloning of 46 candidates that corresponded to full-length sequences within the set of 115 candidates. Effectors are subject to diversifying selection because of the co-evolutionary arms race between host and pathogen proteins [36], [37]. Therefore, we used the presence of amino acid polymorphisms among alignments of deduced protein sequences of *M. persicae* and *A. pisum* ESTs as an additional criterion. Three candidates did not fulfill this criterion and were removed from our candidate set bringing the total to 43 candidates.

We applied a similar data mining approach as described above to 4517 publicly available salivary gland ESTs from *A. pisum*, thereby identifying 24 candidates (Table S1). In the *A. pisum* salivary gland ESTs we predicted only 1751 ORFs, explaining the relatively low number of *A. pisum* candidates. A total of three candidates were found in both *M. persicae* and *A. pisum* datasets (combinations Mp1/Ap1, Mp5/Ap7 and Mp16/Ap4). The remaining 21 non-overlapping *A. pisum* candidates were subjected to BLAST searches (E value<10^−15^) against all available *M. persicae* ESTs to identify putative *M. persicae* homologs. This led to the identification of three *M. persicae* sequences (Mp3, Mp54 and MpC002) that were added to the *M. persicae* candidate effector dataset bringing the total to 46 (Figure 1A, Table S2).

Interestingly, for two candidates, Mp39 and Mp49, no similar sequences were present in the publicly available aphid sequence datasets, including the *A. pisum* genome sequence (Table S2). Also, no homologs of these proteins were identified by BLAST searches against GenBank nucleotide and protein databases (E value<10^−5^). This suggests these proteins may be specific to *M. persicae*. A total of 11 candidates were shared between the independent salivary gland EST datasets from *M. persicae* and *A. pisum* but were not present in gut ESTs from *M. persicae* (Table S2) providing support that the corresponding proteins may share a similar function in both these aphid species. For four candidates matches were found in gut ESTs from *M. persicae*, suggesting these proteins may be derived from salivary gland contaminants in dissected gut tissues and not function uniquely in the salivary gland or saliva. Indeed, gene expression analysis of *Mp51* in various aphid tissues dissected from aphids fed on *N. benthamiana* confirmed that this gene is specifically expressed in the aphid gut (Figure S1). In contrast, candidate effector genes *Mp1*, *Mp2*, *Mp10*, *Mp30*, *Mp42*, *Mp47*, *Mp50* and *MpCOO2*, were expressed in aphid heads and salivary glands but not in aphid guts (Figure S1), suggesting that their corresponding proteins are indeed produced in the salivary glands. Furthermore, Mp1 and MpCOO2 were previously identified in saliva of *M. persicae* using a proteomics-based approach [7] confirming that these two proteins are secreted into aphid saliva.

To investigate the functions of the 46 effector candidates, we amplified the corresponding sequences encoding the mature proteins, without the signal peptide encoding sequences, from *M. persicae* cDNA for cloning (Figure 1B). To preserve the authentic sequence in the 3′ end of the ORF, we designed reverse primers in the 3′ untranslated regions (UTRs) based on EST sequences when possible. Amplicons were cloned in a 35S cassette and corresponding constructs were transformed directly into *Agrobacterium tumefaciens* followed by sequencing (Figure 1B). Two out of the 46 candidates, *Mp7* and *Mp38* could not be amplified from *M. persicae* cDNA. Of the remaining 44 candidates, four (*Mp6*, *Mp17*, *Mp33* and *Mp35*) were represented by two polymorphic forms, with polymorphisms within the mature protein portion. Except for one of the polymorphic *Mp6* sequences, all sequences were identical to those in the *M. persicae* EST databases. To rule out that the polymorphism in *Mp6* was due to PCR errors, we repeated the *Mp6* PCR and sequencing several times on individual aphids with similar results. Both forms of the four polymorphic candidates were cloned resulting in a total of 48 cloned *M. persicae* effector candidates. Functional assays were performed based on transient over-expression in *N. benthamiana* to assess whether the *M. persicae* candidate effectors 1) induce a phenotype *in planta*, 2) suppress PAMP-triggered immunity and 3) affect the ability of *M. persicae* aphids to reproduce (fecundity) (Figure 1B). We assessed fecundity of *M. persicae* lineage RRes (genotype O), which can utilize *N. benthamiana* as a host.

### *M. persicae* candidate effector Mp10 induces chlorosis upon overexpression in *N. benthamiana*

**Figure 2:**
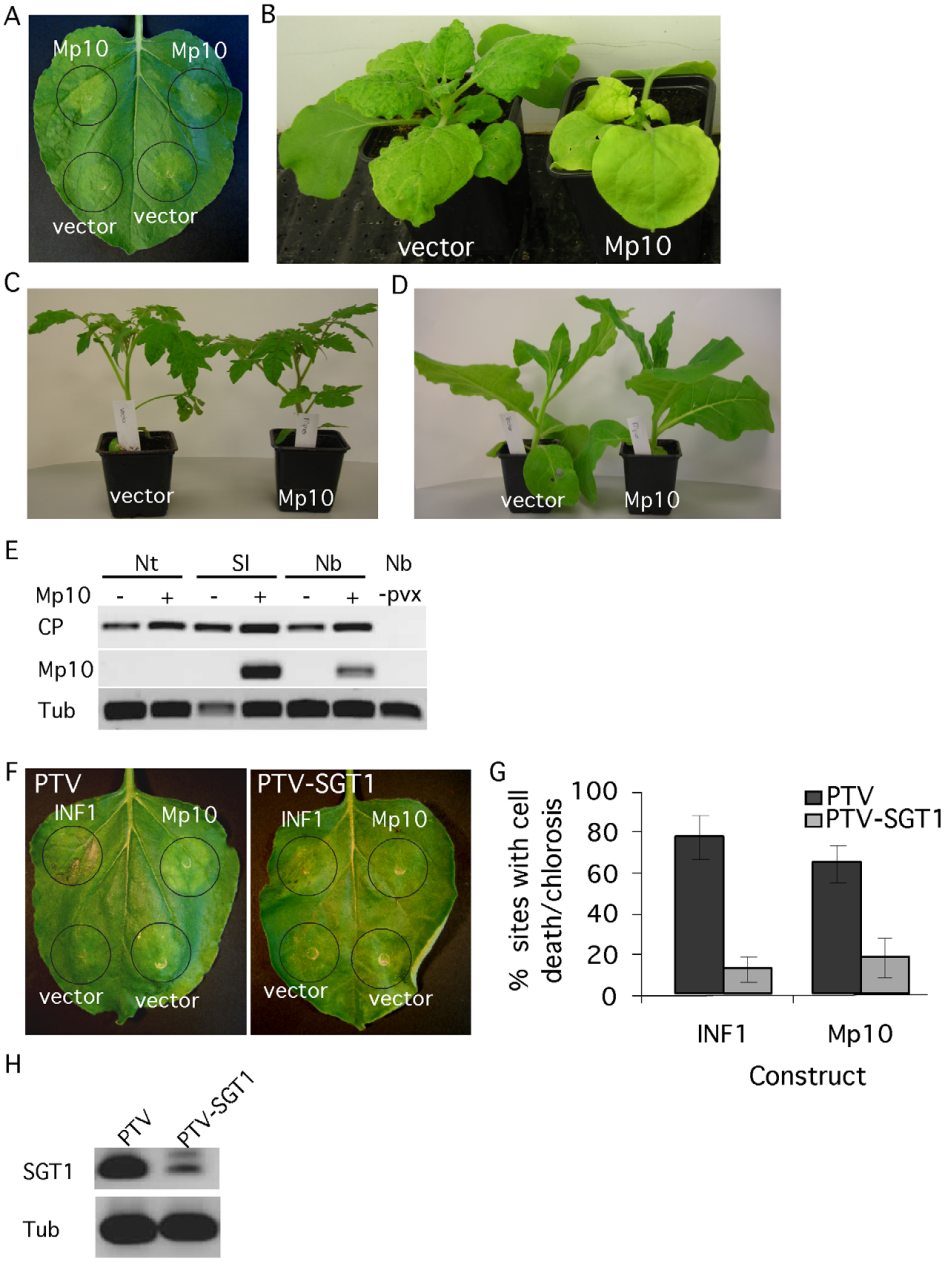
The candidate effector Mp10 induces chlorosis specifically in *N. benthamiana*. (A) Overexpression of 35S-Mp10 by agroinfiltration induces chlorosis in *N. benthamiana*. Symptoms of chlorosis started to appear from 2 days post infiltration (dpi). Photos were taken 4 dpi. (B) PVX-based expression of Mp10 in *N. benthamiana*. Symptoms of chlorosis started to appear from 10 days post wound-inoculation (dpwi). Photos were taken 14 dpwi. (C) PVX-based expression of Mp10 in *Solanum lycopersicum* (tomato). Photos were taken 14 dpwi. D) PVX-based expression of Mp10 in *N. tabacum*. Photos were taken 14 dpwi. (E) Semi-quantitative RT-PCR on RNA from *N. benthamiana* (Nb), *N. tabacum* (Nt) and *S. lycopersicum* (Sl) plants infected with PVX-Δgfp (vector) or PVX-Mp10 as well as non-infected *N. benthamiana* plants (Nb, -pvx). Primers were used to amplify sequences corresponding to the PVX virus coat protein (CP) and Mp10. The plant tubulin gene (Tub) was used as a control for equal RNA levels. Plant tissues were harvested 14 dpwi (F) Over-expression of 35S-INF1 and 35S-Mp10 in *SGT1*-silenced (TRV-SGT1) and control (TRV) *N. benthamiana* plants. Photos were taken 4 dpi. (G) Percentage of infiltration sites showing either INF1 cell death or Mp10 chlorosis 4 dpi on *SGT1*-silenced and control *N. benthamiana* plants. The graphs show the averages calculated from 3 replicated experiments, with 8–10 infiltration sites per individual replicate. Error bars indicate the standard error. H) Semi-quantitative RT-PCR on *SGT1*-silenced and control *N. benthamiana* plants with *SGT1*-specific primers. The plant tubulin gene (Tub) was used as a control for equal RNA amounts.

Several plant pathogen effectors induce a phenotype upon overexpression *in planta*, which may reflect their virulence activity [22]. Hence, we performed transient overexpression of the effector candidates in *N. benthamiana* by agroinfiltration to screen for the induction of phenotypes. Out of the 48, one candidate effector, Mp10, induced chlorosis starting from 2 days post inoculation (dpi) (Figure 2A). In addition, we observed local cell death in a low number of infiltration sites (Figure S2A, S2B, S2C, S2D). The phenotype was not affected by co-expression with the silencing suppressor p19 (Figure S2E). To independently confirm the phenotype, we expressed Mp10 in *N. benthamiana* using a *Potato virus X* (PVX)-based vector (PVX-Mp10). Systemic PVX-based overexpression of Mp10 induced systemic chlorosis in *N. benthamiana* starting at 10 dpi (Figure 2B). This also suggests that the Mp10 response is not dependent on the presence of *Agrobacterium*. To determine whether the response to Mp10 was specific to *N. benthamiana*, we infected *N. tabacum*, *Solanum lycopersicum* (tomato) and *N. benthamiana* plants with PVX-Mp10 in parallel. Starting at around 10 dpi, systemic chlorosis was observed in *N. benthamiana* expressing PVX-Mp10, but not in control PVX-infected plants (Figure 2B). Whereas mosaic symptoms were observed in *S. lycopersicum*, indicative of PVX infection, no Mp10-induced chlorosis was observed (Figure 2C; Figure S3A, S3B). Mp10 expression was confirmed by semi-quantitative RT-PCR in systemically PVX-Mp10 infected leaves of *S. lycopersicum* suggesting that the lack of symptoms is not due to a loss of the Mp10 sequence from PVX-Mp10 (Figure 2E). In contrast, *N. tabacum* plants infected with PVX-Mp10 did not show mosaic symptoms indicative of virus infection, while *N. tabacum* inoculated with PVX alone did (Figure 2D; Figure S2B). No Mp10 expression could be detected in leaves of *N. tabacum* plants inoculated with PVX-Mp10, whereas expression of the viral coat protein was detected, indicating that PVX itself did systemically spread in *N. tabacum* (Figure 2E). In contrast, PVX-Mp42 did spread systemically in *N. benthamiana*, *N. tabacum* and *S. lycopersicum*, indicating that this aphid protein can be systemically expressed in these plant species using PVX (Figure S4). It is possible that PVX-Mp10 may evoke an avirulence response in *N. tabacum* causing the selection of PVX without the Mp10 insert. Loss of foreign gene fragments from the PVX genome has been reported previously and is most likely due to selection pressures forcing virus recombination [38]. The lack of mosaic symptoms in PVX-Mp10-inoculated *N. tabacum* plants is possibly due to the initially low abundance of recombined PVX-virus as compared to the vector control.

The SGT1 protein, an ubiquitin-ligase associated protein, is required for plant cell death responses, including those involved in plant resistance [39]. To investigate whether SGT1 is required for the Mp10 chlorosis response, we generated *SGT1*-silenced *N. benthamiana* plants using *Tobacco rattle virus* (TRV)-based virus-induced gene silencing (VIGS). Silenced plants (treated with TRV-SGT1) and control plants (treated with TRV) (Figure 2H) were infiltrated with *Agrobacterium* strains expressing Mp10 or the positive control INF1, an elicitin from *P. infestans* that induces cell death in control plants, but not in *SGT1*-silenced plants [40]. Both the Mp10-induced chlorosis and the INF1-induced cell death were pronouncedly reduced in the *SGT1*-silenced plants, but not in the TRV-treated control plants (Figure 2F and 2G), indicating SGT1 is required for these chlorosis and cell death responses.

### Candidate effector Mp10 suppresses the flg22- but not the chitin-induced oxidative burst

**Figure 3:**
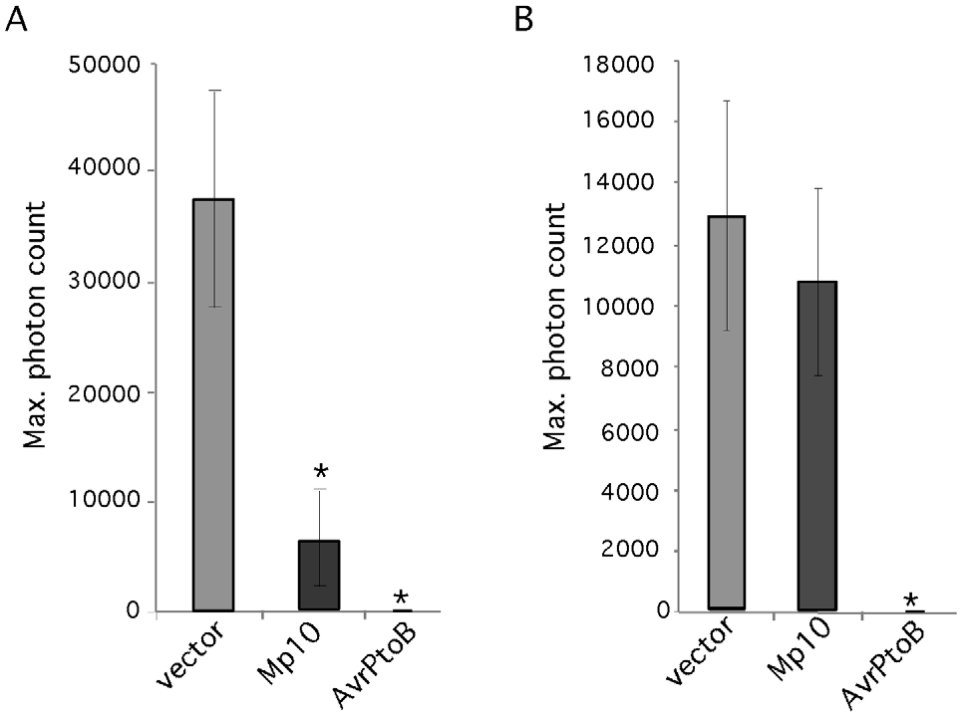
Mp10 suppresses the oxidative burst induced by flg22, but not chitin, in *N. benthamiana*. The induction of reactive oxygen species (ROS) induced by the flg22 and chitin was measured using a luminol-based assay. (A) The ROS response induced by flg22 in *N. benthamiana* leaf discs overexpressing Mp10, AvrPtoB (positive control) and the vector control upon agroinfiltration. The maximum photon count is based on the average of 8 leaf discs. The experiment was repeated 3 times with similar results. Error bars indicate standard error. Asterisks indicate statistical significance compared to the vector control (p≤0.043) (B) The ROS response induced by chitin in *N. benthamiana* leaf discs overexpressing Mp10, AvrPtoB (positive control) and the vector control upon agroinfiltration. The maximum photon count is based on the average of 8 leaf discs. The experiment was repeated 3 times with similar results. Error bars indicate standard error. Asterisks indicate statistical significance compared to the vector control (p≤0.028).

Suppression of PTI induced by PAMPs like flg22 and chitin is a common feature of plant pathogen effectors. To determine whether aphid candidate effectors can suppress PTI, we assessed whether any of our 48 candidates suppressed the oxidative burst response induced by the bacterial PAMP flg22. We decided to screen for suppression of the oxidative burst induced by flg22 only, as this PAMP gives a strong and consistent oxidative burst response in *N. benthamiana*, which is convenient for use in large screens. *N. benthamiana* leaf discs overexpressing the effector candidate genes under control of the 35S promoter were challenged with the flg22 elicitor and the production of reactive oxygen species (ROS) was measured using a luminol-based assay [41]. The bacterial effector AvrPtoB, a suppressor of the flg22-mediated oxidative burst response [42], was included as a positive control. We found that Mp10 suppresses the flg22-induced oxidative burst in leaf discs harvested 2 days post agroinfiltration (three replicated experiments) (Figure 3A), whereas other candidate effectors did not (data not shown). Although the level of suppression by Mp10 was significant compared to that of the empty vector control, it was not as effective as AvrPtoB. We tested whether Mp10 also suppressed the oxidative burst induced by a fungal PAMP, chitin, and found that while Mp10 suppressed the flg22 response, no suppression of the chitin-induced oxidative burst was observed (Figure 3B). Thus, Mp10 specifically suppresses the oxidative burst induced by the PAMP flg22.

### Candidate effectors Mp10, Mp42, and MpC002 alter aphid fecundity on *N. benthamiana*

**Figure 4:**
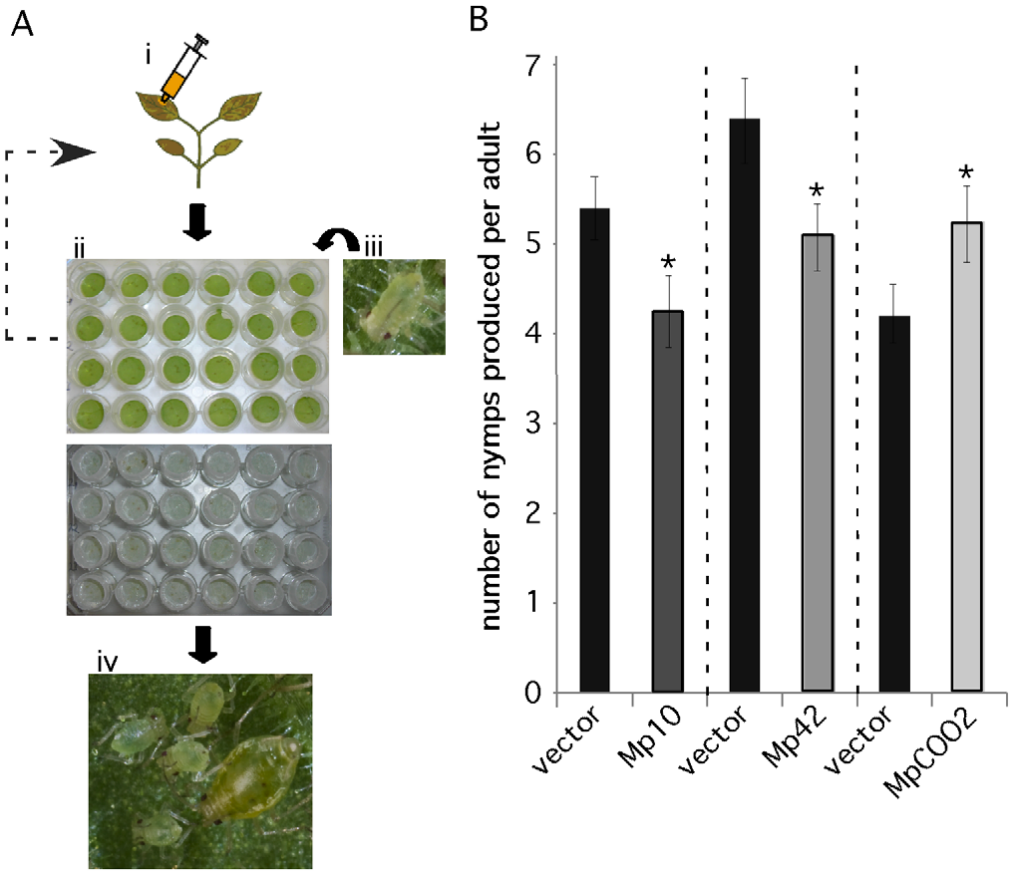
A medium-throughput leaf disc-based assay identifies *M. persicae* effector candidates that affect aphid performance. (A) A novel medium-throughput assay to determine whether *in planta* overexpresssion of effector candidates affects aphid performance. i) Effector candidates are overexpressed in *Nicotiana benthamiana* by agroinfiltration. ii) One day after agroinfiltration leaf discs are harvested from infiltration sites using a cork borer. Leaf discs are placed upside-down on water agar in a 24-wells plate. iii) Four first-instar nymphs are placed on each leaf disc and wells are covered with individual mesh caps. Every six days these four aphids are moved to fresh leaf discs overexpressing the effector candidates. iv) Nymph production is assessed up to 17 days after placing first-instar nymphs on the leaf discs on day 1. (B) Overexpression of Mp10 and Mp42 reduces aphid nymph production (fecundity) and overexpression of MpC002 increases aphid nymph production. For each effector candidate, agroinfiltrations and aphid assays were performed side-by-side with the vector control (vector). Graphs show the average number of nymphs produced per adult based on 3 replicated experiments, each consisting of 6 replicated leaf discs per candidate effector construct (n = 18). Error bars indicate the standard error. Asterisks indicate statistical significance compared to the vector control based on a one-way ANOVA (Mp10: p≤0.026, Mp42: p≤0.036 and MpCOO2: p≤0.038).

We developed a medium-throughput 24-well plate assay to assess *M. persicae* fecundity on *N. benthamiana* leaves transiently overexpressing the 48 candidate effectors (Figure 4A). Leaf discs were harvested from infiltrated leaves one day after agroinfiltration and placed upside down on water agar in 24-well plates. Four first-instar nymphs were placed on each leaf disc and the plate was incubated up-side-down under a light source. Aphids were moved every 6 days to plates with freshly infiltrated leaf discs, as expression levels of green fluorescent protein (GFP) in leaf discs were constant during 6 days and then tapered off (Figure S5). The aphids placed initially on the leaf discs generally started producing nymphs after about 10–11 days. Nymph production (fecundity) was assessed on day 12, 14 and 17 by counting and removing newly produced nymphs on each leaf disc. The total nymph production per adult was calculated and compared among the treatments and GFP and vector controls.

In our initial screens, in which candidate effector constructs were infiltrated on different leaves and not always side-by-side with the vector control, we identified 14 candidates that either enhanced or reduced aphid fecundity by one time the standard error compared to the empty vector (EV) control (Figure S6). To confirm the effect on aphid fecundity of these 14 candidates, we conducted additional assays in which the candidates were infiltrated side-by-side with the vector control (EV) on the same leaves. Two candidates, Mp10 and Mp42, reduced aphid fecundity in three repeated confirmation assays compared to the vector control (Figure 4B). In addition, one candidate, MpC002, enhanced aphid fecundity in three repeated confirmation assays compared to the vector control (Figure 4B). Transient overexpression of Mp10 did not induce chlorosis in leaf discs (Figure S7) or leaves that were detached from the plant 24hrs after infiltration (data not shown). Thus, leaves need to be attached to the plant for chlorosis to occur and the chlorosis itself was therefore not likely responsible for the observed reduction in aphid performance. In summary, we have developed a novel assay to screen for effects of *in planta* expressed aphid salivary proteins on aphid performance and thereby identified three candidates that potentially function as effectors by eliciting plant defenses or promoting aphid infestation of host plants.

### Homology searches of Mp10, Mp42, and MpC002

**Figure 5:**
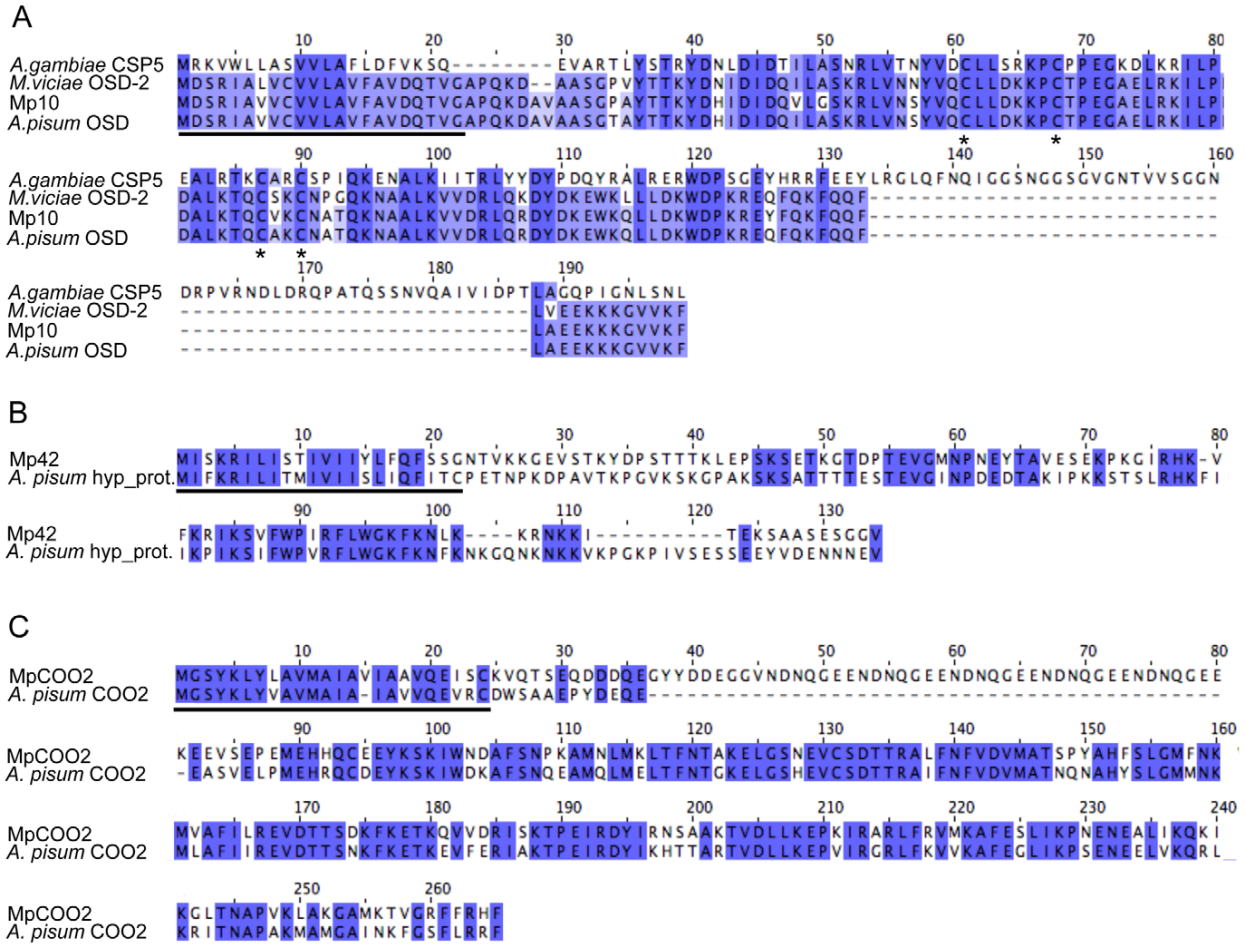
Amino acid alignments of *M. persicae* effector candidates that alter aphid fecundity. Black lines indicate the predicted signal peptide sequences. (A) Alignment of Mp10 with similar sequences from the aphid species *Acyrthosiphon pisum* (GenBank accession NP_001119652.1), *Megoura viciae* (GenBank accession CAG25435.1), and the mosquito species *Anopheles gambiae* (GenBank accession XP_317401.4). Asterisks indicate conserved cysteine residues. (B) Alignment of Mp42 with a similar sequence from *A. pisum* (GenBank accession XP_001948510). (C) Alignment of MpC002 with a similar sequence from *A. pisum* (GenBank accession XP_001948358.1).

To determine whether the candidates that alter aphid fecundity, (i.e. Mp10, Mp42, and MpC002) share similarity to proteins of known or predicted function, we performed BLAST searches against the GenBank non-redundant (nr) protein database (E value<10^−5^). One of the three candidates, Mp10 showed homology to an insect protein of predicted function, the olfactory segment D2-like protein (OS-D2-like protein). The OS-D2-like protein is a member of a family of chemosensory proteins in aphids that contain the conserved cysteine pattern CX_6_CX_18_CX_2_C [43]. Mp10 also shows similarity to chemosensory proteins (CSPs) from other insects (E value<10^−5^), including the CSP5 protein from the mosquito *Anopheles gambiae* (Figure 5A). The four cysteines in Mp10 are conserved among different members of the CSP family [44], [45] (Figure 5A). Among the aphid sequences similar to Mp10, polymorphisms are predominantly present after the predicted signal peptide sequence, in the mature protein region. For Mp42 and MpC002, similar sequences were identified in the genome sequence of the aphid species *A. pisum* only, but these proteins have no similarities to proteins with known functions. Alignment of Mp42 to a putative *A. pisum* homolog shows strong sequence divergence especially in the mature protein regions (Figure 5B). Finally, alignment of MpC002 to *A. pisum* C002 shows sequence divergence consisting of both amino acid polymorphisms and a 45 amino acid gap in *A. pisum* C002 after the predicted signal peptide sequence (Figure 5C). The presence of polymorphisms mainly in the mature protein regions may reflect that the functional domains of these proteins have diversified due to distinct selective pressures.

## Discussion

Aphids, like other plant parasites, deliver repertoires of proteins inside their hosts that function as effectors to modulate host cell processes. These insects most likely secrete effectors into their saliva while progressing through the different plant cell layers during probing and feeding. The identification and characterization of these proteins will reveal new insights into the molecular basis of plant-insect interactions. Here, we have described a functional genomics pipeline to identify *M. persicae* effector candidates as well as various assays to determine whether the candidates share features with plant pathogen effectors. Using this approach, we identified three candidate effectors, Mp10 and Mp42, MpC002 that modulate host cell processes and affect aphid performance.

The induction of chlorosis and local cell death by Mp10 can reflect a genuine effector activity of this aphid salivary protein. Ectopic expression of bacterial TTSS as well as filamentous plant pathogen effectors can affect host immunity and induce a variety of phenotypes in plants, ranging from chlorosis to necrosis [22], [28]. Both the *P. syringae* type III effectors AvrB [30] and HOPQ-1 [46] induce chlorosis and for AvrB this activity is plant genotype specific [47]. No Mp10 induced chlorosis was observed in tomato despite expression levels of PVX-Mp10 that were comparable to *N. benthamiana*. This suggests that the Mp10 response was specific for *N. benthamiana*. Interestingly, PVX-Mp10 was unable to infect *N. tabacum*, suggesting this protein may induce an unknown defense mechanism that is effective against PVX-Mp10.

There are several possibilities that may explain the Mp10 phenotype in a biologically relevant context. The first possibility is that the artificially high expression of Mp10 could lead to the induction of the chlorosis/local cell death phenotype and therefore this response could be an artifact of the *Agrobacterium*-mediated overexpression assay. However, in this case we would expect that the induction of chlorosis and local cell death by Mp10 would be more widespread in various plant species, and would also be observed in *N. benthamiana* leaf discs or detached leaves. Another possibility is that the high expression of Mp10 could lead to excessive targeting of the operative target as well as other host proteins leading to an exaggeration of the true virulence activity [22]. Finally, the induction of chlorosis and local cell death could reflect avirulence activity of Mp10. Feeding of *M. persicae* is known to induce chlorosis and premature leaf senescence in plants, and this response is related to PAD4-mediated defense responses [48]. Therefore, Mp10 may exhibit an avirulence activity specifically in *Nicotiana* spp resulting in chlorosis and local cell death. The induction of chlorosis in *N. benthamiana* by *P. syringae* effector AvrB is thought to be due to weak activation of TAO1, an NBS-LRR protein, and requires the plant-signaling component Rar1 [49]. We found that chlorosis induction by Mp10 requires the co-chaperone SGT1, which is required for activation of NBS-LRR proteins and plant resistance responses [39]. Therefore, Mp10 may activate an NBS-LRR resistance protein resulting in ETI (further discussed below).

We also found that Mp10 suppressed the ROS response induced by flg22, suggesting that suppression of PTI may be a feature shared by plant pathogens and insects. Possibly, the flg22-induced signaling pathway may not be specific to bacteria as other (non-bacterial) PAMPs can induce this pathway. Also, plants may have a PTI pathway(s) that is induced by an unknown insect PAMP(s) and partially overlaps with the signaling pathway induced by flg22. To date the role of perception of PAMP-like molecules in plant-insect interactions remains elusive. However, chitin is a major structural component of the insect cuticle. Degradation of chitin by plant chitinases generates fragments that induce PTI [50]. Whether the chitin in the insect cuticle is degraded to induce plant defenses during interaction with host plants remains to be investigated. It has been hypothesized that sheath saliva protects the insect stylets, which mainly consist of chitin, from triggering plant defenses [51]–[53], potentially including PTI. Recent studies showed that insect saliva of both chewing insects [54] and aphids [55] contains elicitors that induce defense responses in host plants. The nature of these elicitors and their role in triggering PTI are unknown. Despite the lack of an understanding of the role in perception of PAMP-like molecules in plant-insect interactions, our data suggest that an aphid salivary protein, Mp10, can interfere with a specific PAMP response in a *M. persicae* host plant. It is therefore possible that Mp10 is a genuine suppressor of PTI. Alternatively, the overexpression of Mp10 may perturb a signaling component in the PTI pathway that is required for recognition of flg22. As Mp10 induces weak chlorosis starting from 2 dpi, it is possible that this response itself is responsible for loss of the oxidative burst response to PAMPs. However, the Mp10 chlorosis response does not interfere with the oxidative burst triggered by chitin. This suggests that the induction of chlorosis itself may not be sufficient to block the oxidative burst induced by flg22, but that Mp10 specifically interferes with the flg22-triggered signaling cascade.

Despite the suppression of the flg22-mediated oxidative burst by Mp10, its overexpression in *N. benthamiana* reduced aphid fecundity. A plausible explanation for this contradictory observation is that Mp10 may activate an NBS-LRR resistance protein resulting in ETI, thereby reducing aphid performance. Thus, the recognition of Mp10, potentially through ETI, in *Nicotiana* spp may mask the true virulence activity of this protein. If true, this recognition may be suppressed by other effectors during plant-aphid interactions so that Mp10 can exhibit its virulence function.

The leaf disc assay allowed us to generate vast amounts of functional data and directly implicated three effector candidates in plant-aphid interactions. The differences in aphid fecundity observed in our screens were quite variable, requiring replication of experiments. Despite the variation, Mp10, Mp42, and MpC002 showed consistent effects on aphid fecundity throughout the individual replicates (data not show). The fecundity was affected by Mp10, Mp42, and MpC002 by around 1–1.5 nymph produced per adult over a nymph production period of about 6 days. Although these differences may seem small, they are expected to have a large impact on the population size of aphids. Furthermore, *M. persicae* does not perform as well on *N. benthamiana* as it does on other hosts, such as *Arabidopsis thaliana*. Despite the low reproduction level on *N. benthamiana*, the fecundity differences found in our screens are similar to those observed over a 2-day period on *A. thaliana* in a study by Pegadaraju et al. [56] which shows that overexpression of PAD4 reduced aphid fecundity by about 1.5 nymphs per adult. The number of candidate effectors with an effect on aphid fecundity identified in this study may have been limited by our approach. For example, when the amount of an effector secreted by the aphid is sufficient to modulate host cell processes to promote feeding, *in planta* overexpression may not necessarily further enhance this effect. Also, there could be differences in plant responses to aphids in leaf discs versus whole plants as certain plant responses to aphids may require an intact plant transport system. Despite these limitations, the development of a novel leaf disc-based assay allowed us to identify three effector candidates from the aphid species *M. persicae*.

Out of the three candidates that affect aphid fecundity in the leaf-disc assays, only Mp10 shows homology to a protein of predicted function, namely OS-D2, a member of a family of predicted chemosensory proteins. Insect chemosensory proteins (CSP) are thought to be involved in olfaction and gustation. Indeed, several CSPs have been specifically found in chemosensory organs and are predicted to function in chemoperception [43], [57], [58]. However, for some members of this large protein family functions have been identified in insect development [59] and leg regeneration [60], suggesting that CSPs may have divergent functions. This is further supported by gene expression studies, which show that some CSPs are specifically expressed in antenna [61] or mouthparts [62], whereas others are expressed throughout the insect [63]. CSPs are thought to bind small molecules, such as fatty acids, and for some members of this protein family there is evidence that they bind to pheromones [64], [65]. In the aphid species *Megoura viciae* a Mp10 homolog was found to be expressed in aphid heads without antenna, indicating that it is not an antenna specific CSP [43]. Interestingly, in mosquitos, members of a family of odorant binding-related proteins, also with predicted functions in olfaction and gustation, are secreted into host cells to manipulate host physiology by for example scavenging host amines [66]. Counteracting host amines has evolved in various blood-feeding insects independently through adaptation of members of the lipocalin or odorant-binding protein families [66]. It is possible that also in plant feeding insects, proteins predicted to be involved in chemosensing are actually involved in early plant host recognition and plant host cell manipulation.

For Mp42 and MpC002 no homology was found to proteins of known or predicted function. This is not surprising as most plant pathogen effectors described to date do not show similarity to proteins of known function based on amino acid alignments. The reduction in aphid performance upon overexpression of Mp42 could reflect that Mp42 induces defense responses against aphids in the plant. In contrast, the enhancement of aphid fecundity by MpC002 suggests that this protein may exhibit an effector activity to promote aphid infestation. Indeed, the *A. pisum* homolog of MpC002, ApC002, has been implicated in aphid feeding [5]. Interestingly, ApC002 is secreted into plant tissues during aphid feeding and silencing of *ApC002* gene expression reduces aphid survival on plants, but does not affect when aphids feed from diet [67]. However, whether *A. pisum* performs better upon overexpression of *C002 in planta* is not known. Our data suggest that the MpC002 homolog may exhibit a similar role in *M. persicae*, and that this protein is important during plant-aphid interactions. Future studies will be aimed at further characterizing these candidates to identify their plant targets and the molecular processes they perturb.

## Methods

### Sequence databases

We downloaded the following datasets in November 2008 from GenBank for bioinformatics analyses. A total of 3233 *M. persicae* salivary gland ESTs, 27868 *M. persicae* ESTs (all available ESTs), and 2558 *M. persicae* gut ESTs [34], as well as 4517 *A. pisum* salivary gland ESTs (GenBank accessions DV747494-DV752010). For similarity searches against the *A. pisum* genome sequence, we obtained the whole shotgun genome sequence scaffolds from GenBank (accessions EQ110773-EQ133570) in May 2010.

### Bioinformatics analyses

The pipeline for the identification of *M. persicae* candidate effectors was developed as follows. The 3233 salivary gland ESTs from *M. persicae* were subjected to ORF calling. More specifically, we performed translations of all possible ORFs of 70+ amino acids, defined by an ATG to stop or an ATG to the end of a sequence, from both strands of the cDNA. We then applied the SignalP v 3.0 program [35] to predict the presence of signal peptides in the amino acid sequences with an HMM score cut-off value of >0.9 and a predicted cleavage site within the amino acid region 1–30. As some predicted secreted proteins were represented multiple times within the *M. persicae* salivary gland EST dataset, we used BLASTP searches to remove redundant sequences. Alignments were inspected manually and sequences that showed >95% identity throughout most of the alignment with an E value<10^−10^ were classified as being redundant. To remove sequences that in addition the signal peptide also contained a transmembrane domain we used TMHMM v.2.0. The remaining sequences were searched using TBLASTN (E value<10^−5^) against all *M. persicae* and *A. pisum* ESTs in our datasets as well as the *A. pisum* genome sequence to assess whether they encoded full-length proteins. Criteria for selecting full-length sequences were: 1) the presence of a conserved start and stop site in ESTs within the alignments; 2) the absence of a methionine within the alignments upstream of the methionine predicted to be the start of the ORF; 3) similarity to a predicted full-length *A. pisum* protein, when available. The remaining predicted secreted protein sequences were then assessed for the presence of polymorphisms within the alignments described above. Sequences not showing any sequence variation in alignments with *M. persicae* sequences and that contained up to one amino acid difference in alignments of the mature protein regions with *A. pisum* sequences were removed from the candidate list.

The 4517 salivary gland ESTs from *A. pisum* were analyzed with the same procedures except that no analyses was performed for the presence of polymorphisms. The amino acid sequences of the predicted secreted proteins (Table S1) were searched using BLASTP (E value of <10^−5^) against the amino acid sequences of the *M. persicae* candidates to identify overlap in the datasets. *A. pisum* candidates without a hit were then searched using TBLASTN against all available *M. persicae* ESTs (E value of <10^−5^) to identify *M. persicae* predicted secreted proteins with sequence similarity. The *M. persicae* candidates identified using our pipeline and subjected to cloning were designated MpC002, Mp1-12, Mp14-17, Mp19-24, Mp28-33, Mp35-37, Mp39-47, Mp49-51, Mp53-54, wherein Mp stands for *M. persicae* (Table S2).

### Aphids

The *M. persicae* colony of lineage RRes (genotype O) was maintained in cages on *N. tabacum* plants. Cages were located in a contained growth room at 18°C under 16 hours of light.

### Microbial strains and growth conditions

*A. tumefaciens* strain GV3101 was used in molecular cloning and agroinfiltration experiments and were routinely cultured at 28°C in Luria-Bertani (LB) media using appropriate antibiotics [68]. All bacterial DNA transformations were conducted by electroporation using standard protocols [68].

### Cloning of Mp candidates

Primers were designed for amplification of sequences corresponding to the ORFs encoding the mature proteins (after the signal peptide encoding sequences) (Table S3). To confirm the 3′ end of the ORFs, we designed, where possible, the 3′-primer in the 3′UTR sequence. Sequences were amplified from *M. persicae* cDNA using Phusion polymerase (Finnzymes) and ligated into *Spe*I/*Bam*HI, *Spe*I/*Bgl*II or *Bgl*II/*Bam*HI digested pCB302-3 vector [69] to generate 35S-constructs. To assess whether sequences were polymorphic within the *M. persicae* clonal lineage used in our studies, we performed sequence analyses of 4 clones per construct. To generate constructs for PVX-based expression, we amplified sequences encoding mature ORFs and ligated these into *Cla*I/*Not*I digested pGR106 vector. The PTV vectors used in this study have been described previously [40].

### Gene expression analyses by semi-quantitative RT-PCR

Aphids were dissected in PBS and tissues stored in RNA later. We collected 25 salivary glands, 10 guts, 5 heads and 5 whole aphids. RNA extractions were performed with the NucleoSpin RNA XS kit (Macherey-Nagel, Germany). cDNA was synthesized from 80 ng total RNA per sample using expand reverse transcriptase (Roche Diagnostics Ltd). RT-PCR was performed with gene specific primers for each effector candidate indicated in Table S3. MpActin primers were used as a control for equal cDNA template amounts.

For RT-PCR on plant tissues, 50 mg leaf tissue was ground in liquid nitrogen and RNA was extracted with the RNeasy Plant minikit (Qiagen). cDNA was synthesized from 500ng DNase treated RNA and subjected to PCR reactions with primer pairs Mp10-pvx-F/R and Mp42-pvx-F/R (Table S3) for amplification of Mp10 and Mp42 expressed in PVX, respectively. For amplification of the PVX coat protein we used primer pair PVX-CP-F/R and for amplification of plant tubulin we used the primer pair Tub-F/R (Table S3). Primers used for RT-PCR on RNA extracted from SGT- and HSP90-silenced plants were described elsewhere [26].

### PVX agroinfection and agroinfiltration assays

Recombinant *A. tumefaciens* strains were grown as described elsewhere [70] except that the culturing steps were performed in LB media supplemented with 50 µg/mL of kanamycin. Agroinfiltration experiments were performed on 4–6 week-old *N. benthamiana* plants. Plants were grown and maintained throughout the experiments in a growth chamber with an ambient temperature of 22°–25°C and high light intensity.

For transient overexpression of candidate effectors by agroinfiltration, leaves of *N. benthamiana* were infiltrated with *A. tumefaciens* strain GV3101 carrying the respective constructs at a final OD_600_ of 0.3 in induction buffer (10mM MES, 10mM MgCl_2_, 150 µM acetosyringone, pH = 5.6).

For agroinfection assays, cotelydons of *N. benthamiana*, *N. tabacum* (cv Petite Gerard) or *S. lycopersicum* (MoneyMaker) were wound-inoculated with candidate effector clones using P200 pipette tips. Each strain was assayed on 2 replicated plants. As a control, plants were wound-inoculated with *A. tumefaciens* strains carrying pGR106-Δgfp [26]. Systemic PVX symptoms were scored 14 days post inoculation.

### TRV-induced gene silencing

We performed gene silencing as described elsewhere [40]. *A. tumefaciens* suspensions expressing the binary TRV-RNA 1 construct, pBINTRA6, and the TRV-RNA2 vector, PTV00 or PTV-SGT1 were mixed in 1∶1 ratio (RNA1- RNA2) in induction buffer (final OD600 is 0.6). Leaves were challenged with *Agrobacterium* strains carrying 35S-Mp10 and 35S-INF1 or the 35S vector.

### Aphid fecundity assays in 24-well plates

We developed a medium-throughput 24-well assay to test whether overexpression *in planta* of effector candidates affects aphid nymph production rates. For this purpose, we overexpressed the candidates (35S-constructs) by agroinfiltration in *N. benthamiana* at a final OD600 of 0.3. One day after infiltration, leaf discs were collected using a cork borer (No. 7) from the infiltration sites and placed upside-down on top of 1ml water agar in 24-well plates. A total of 6 infiltration sites, from 6 different leaves, were used per construct and a total of 4 different constructs per 24-well plate. In initial screens, we infiltrated multiple sets of 4 candidate effectors at the same time, with one set including the vector and GFP controls (two candidate effectors plus the two controls). The 4 candidates within a set were infiltrated side-by-side on the same 6 leaves. Leaf discs from each set of candidates were placed in one 24-well plate (6 discs times 4 candidates). For the confirmation assays, we performed infiltrations of each candidate effector with the vector control side-by-side on the same 6 leaves, and leaf discs were placed in one 24-wells plate. On each leaf disc, we placed 4 *M. persicae* first-instar nymphs. The wells in the plate were individually sealed off using a cap of a 5ml BD Falcon round bottomed test tub with the top of the cap removed and covered with mesh. After 6 days, the nymphs were moved to a new 24-wells plate with fresh leaf discs infiltrated with the candidate effector constructs. Another 6 days later, the now adult aphids were again moved to a new 24-well plate with freshly infiltrated leaf discs. The numbers of adults (initially first-instar nymphs) were counted 6, 12, 14 and 17 days after setting up the first 24-wells plate and the number of newly produced nymphs were counted on day 12, 14 and 17. The newly produced nymphs were removed from the wells during counting. Wells wherein all 4 aphids that were initially placed on the discs died were taken out of the analyses. To calculate the production of nymphs per adult aphid, we calculated the average number of nymphs produced per adult by combining the average production rates throughout the experiment. These average production rates were obtained by dividing the number of nymphs on day 12 by the number of adults on day 6 (calculated per well), dividing the number of nymphs on day 14 by the number of adults on day 12, and dividing the number of nymphs on day 17 by the number of adults on day 14. To obtain the total average production rate, we calculated the sum of the average production rates for days 12, 14 and 17.

### Measurements of reactive oxygen species

*N. benthamiana* leaf discs transiently overexpressing the effector candidates were subjected to a luminescence-based assay [41]. Leaf discs were floated overnight in 200ul water in a 96-well plate. The production of ROS was measured after replacing the water with a solution of luminol (20uM) and horseradish peroxidase (1ug) supplemented with either flg22 peptide (100nM) or chitin (100 µg/ml). Luminescence was measured using a Varioskan Flash plate reader. A total of 8 discs per construct, obtained from 4 different infiltration sites, were used per replicate. Assays with flg22 to screen the 48 candidates for suppression activity were repeated two times. The assays with chitin and flg22 were repeated three times.

### Statistical analyses

All statistical analyses were conducted using Genstat 11. ROS assay was analysed using a two-sample t-test. Leaf discs fecundity assays were analysed using one-way ANOVA with “construct” as the treatment and “repeat” as the block. Data was checked for approximate normal distribution by visualising the residuals.

## Supporting Information

Figure S1 Gene expression analyses of candidate effectors in various aphid tissues. RT-PCR was performed on cDNA prepared from whole aphids fed on *N. benthamiana*, dissected heads, guts, salivary glands and on H_2_O (control). Candidates were amplified using gene specific primers. Actin primers were used as a control for equal template amounts.
(5.23 MB TIF)

Figure S2 Mp10 induces weak local cell death in *N. benthamiana*. (A–D) Symptoms of *N. benthamiana* agroinfiltration sites expressing the 35S empty vector (control) or Mp10 under bright and ultraviolet (UV) light. Symptoms induced by the control (A) and Mp10 (B) were analyzed under a dissecting microscope. Accumulation of autofluorescent phenolic compounds associated with local cell death induced Mp10 (D), but not the control (C) were visualized under ultraviolet (UV) light (480/40 nm excitation filter; 510 barrier nm). Photographs were taken 5 days post infiltration. The black arrow heads indicate foci associated with autofluorescent phenolic compounds as a result of local cell death. (E) Percentages of infiltration sites showing induction of local macroscopic cell death upon expression of the Mp10 in *N. benthamiana* plants. Leaves were agroinfiltrated with *Agrobacterium* strains carrying 35S-Mp10 or PVX-Mp10 in the presence or absence of strains carrying p19 at an OD600 of 0.3 or 0.6. NS indicates no symptoms, CHL indicates chlorosis and CHL+CD indicates cell death. Symptoms were scored 4 days post infiltration. The average number of infiltration sites was based on 3 replicated experiments (n = 8 sites per experiment). Error bars indicate the standard error.
(2.59 MB TIF)

Figure S3 Symptoms of PVX-Mp10 infected *Solanum lycopersicum* (tomato) and *N. tabacum* plants. (A) Symptoms on a tomato plant infected with PVX-Δgfp (control) (left panel) and PVX-Mp10 (right panel). (B) Symptoms on a *N. tabacum* plant infected with PVX-Δgfp (control) (left panel) and PVX-Mp10 (right panel). Pictures were taken 14 days after inoculation.
(8.70 MB TIF)

Figure S4 PVX-based expression of *Mp42* in various plant species. Leaf tissues from *N. benthamiana* (Nb), *N. tabacum* (Nt), and *Solanum lycopersicon* (Sl) were collected for RNA extractions 14 days post wound-inoculation (dpwi). For semi-quantitative RT-PCR primers were used to amplify sequences corresponding to the PVX virus coat protein (CP) and Mp42. The plant tubulin gene (Tub) was used as a control for equal RNA amounts.
(0.79 MB TIF)

Figure S5 Expression of GFP in *N. benthamiana* leaf discs placed on water agar in a 24-well plate. Leaves were collected 24 after agroinfiltration with *Agrobacterium* strains expressing GFP and placed on top of water agar in a 24 wells plate. Leaf discs were collected every 24 hours from 1 to 7 days post infiltration (DPI) and ground in SDS-PAGE sample loading buffer to analyze the accumulation of GFP by western blotting with a GFP antibody. As a negative control (C) a 1-day old non-infiltrated *N. benthamiana* leaf disc was used. Ponceau S staining (PS) showed equal loading.
(0.26 MB TIF)

Figure S6 Overexpression of *M. persicae* candidate effector in *N. benthamiana* alters aphid reproductive performance (fecundity). Using the leaf disc-based assay, a set of 48 candidate effectors was expressed in *N. benthamiana* by agroinfiltration to screen for effects on aphid fecundity. Red dotted lines mark sets of candidates that were screened in parallel experiments. EV indicates the vector control and GFP indicates the GFP control. Nymph production was counted over a 17-day period. The average number of nymphs produced per adult was based on 3 replicated experiments. Error bars indicate the standard error. Asterisks indicate Mp candidates that were further tested in confirmation assays.
(0.05 MB PDF)

Figure S7 Symptoms of *N. benthamiana* infiltration sites expressing Mp10 during the leaf disc 24-well plate assay. Photo was taken 5 days after infiltration.
(0.84 MB TIF)

Table S1 List of candidate effectors of the pea aphid (*Acyrthosiphon pisum*).
(0.04 MB XLS)

Table S2 List of candidate effectors of the green peach aphid (*M. persicae*).
(0.06 MB XLS)

Table S3 Primer table.
(0.11 MB DOC)
