# Supplementary figures and images for: A Functional Genomics Approach Identifies Candidate Effectors from the Aphid Species Myzus persicae (Green Peach Aphid)
Source: PLoS Genet. 2010 Nov 18;6(11):e1001216. doi: 10.1371/journal.pgen.1001216 (PMC2987835; doi:10.1371/journal.pgen.1001216)

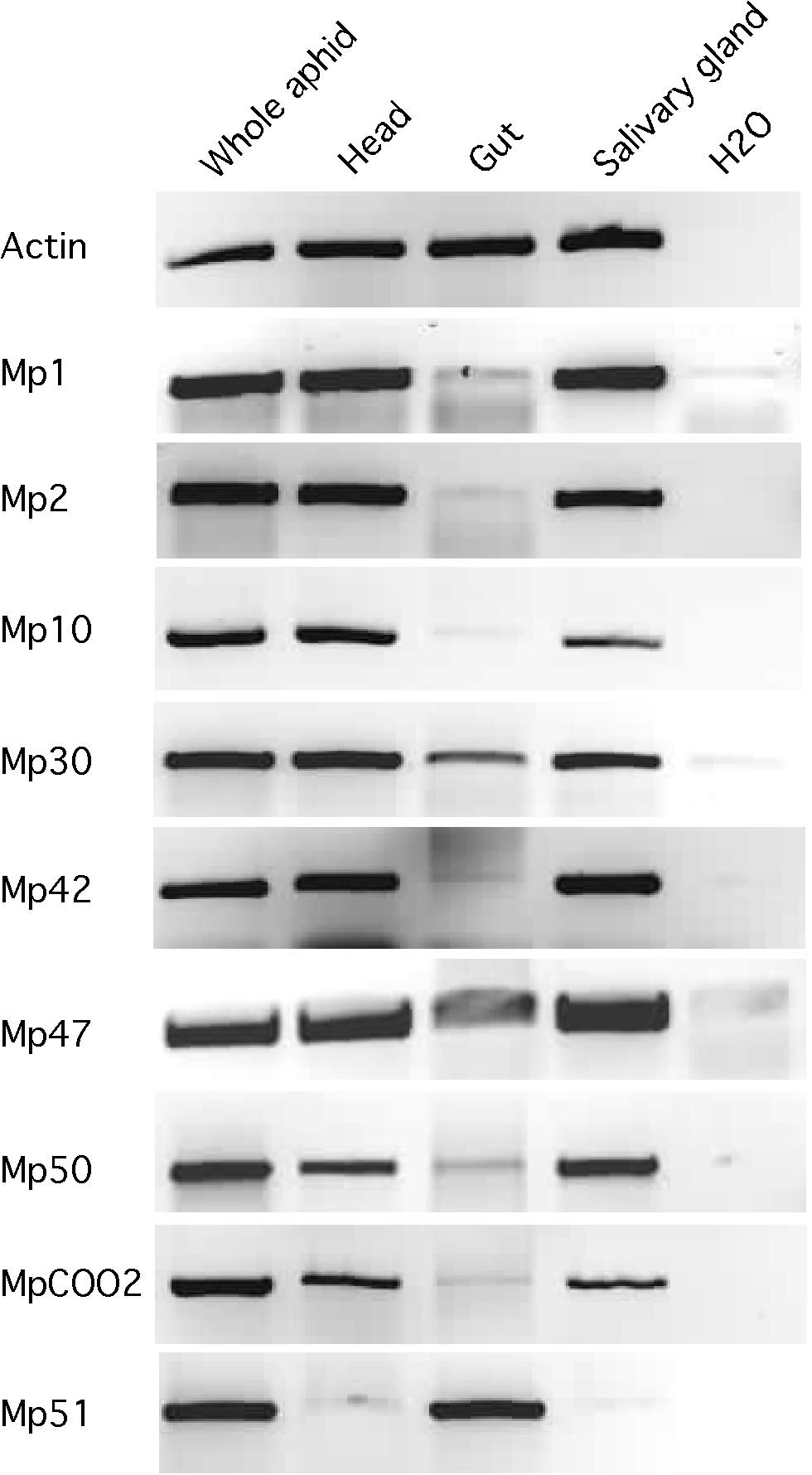

Supplement: Figure S1 — Gene expression analyses of candidate effectors in various aphid tissues. RT-PCR was performed on cDNA prepared from whole aphids fed on N. benthamiana, dissected heads, guts, salivary glands and on H2O (control). Candidates were amplified using gene specific primers. Actin primers were used as a control for equal template amounts. (5.23 MB TIF) [file pgen.1001216.s001.tif]

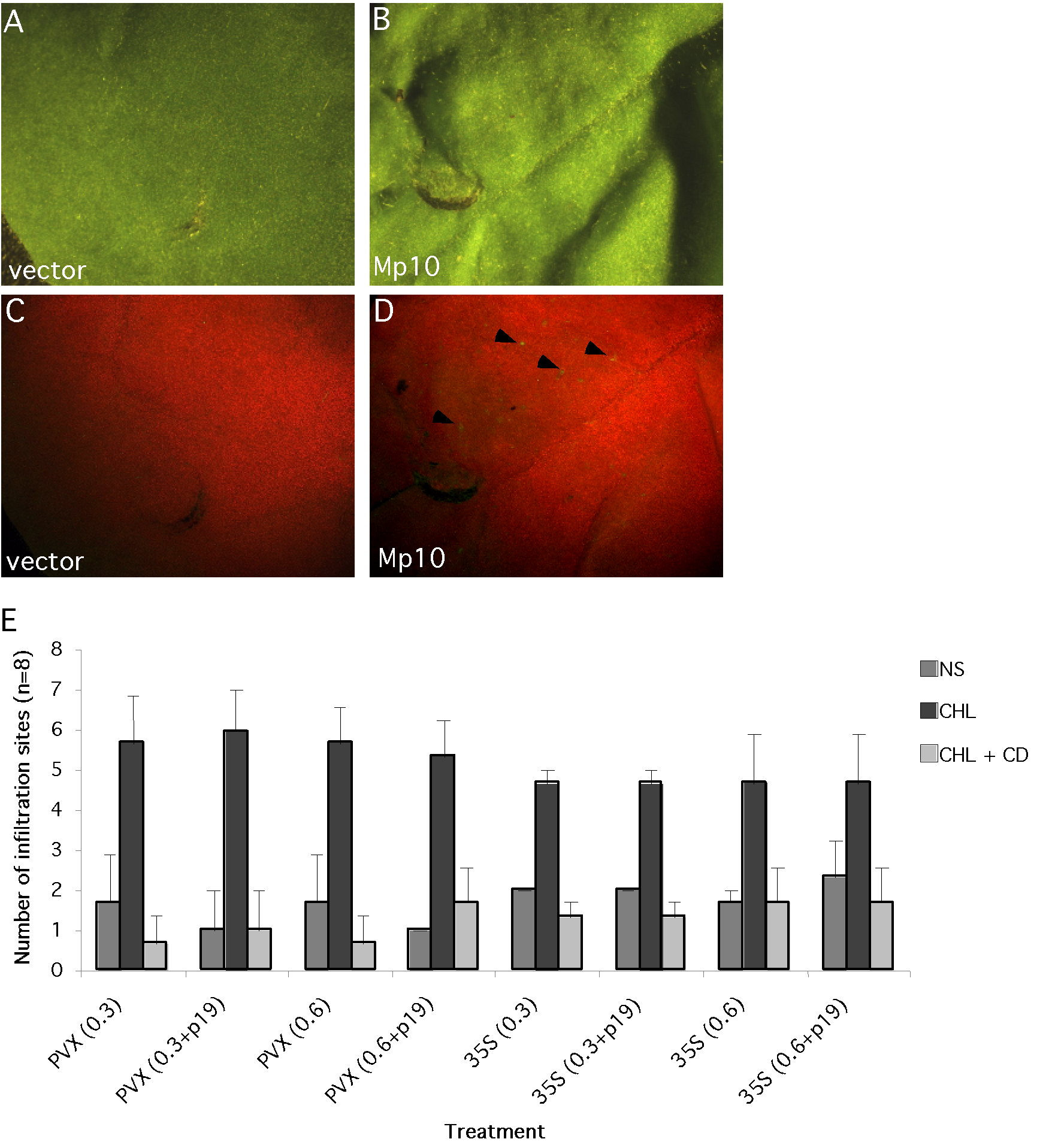

Supplement: Figure S2 — Mp10 induces weak local cell death in N. benthamiana. (A–D) Symptoms of N. benthamiana agroinfiltration sites expressing the 35S empty vector (control) or Mp10 under bright and ultraviolet (UV) light. Symptoms induced by the control (A) and Mp10 (B) were analyzed under a dissecting microscope. Accumulation of autofluorescent phenolic compounds associated with local cell death induced Mp10 (D), but not the control (C) were visualized under ultraviolet (UV) light (480/40 nm excitation filter; 510 barrier nm). Photographs were taken 5 days post infiltration. The black arrow heads indicate foci associated with autofluorescent phenolic compounds as a result of local cell death. (E) Percentages of infiltration sites showing induction of local macroscopic cell death upon expression of the Mp10 in N. benthamiana plants. Leaves were agroinfiltrated with Agrobacterium strains carrying 35S-Mp10 or PVX-Mp10 in the presence or absence of strains carrying p19 at an OD600 of 0.3 or 0.6. NS indicates no symptoms, CHL indicates chlorosis and CHL+CD indicates cell death. Symptoms were scored 4 days post infiltration. The average number of infiltration sites was based on 3 replicated experiments (n = 8 sites per experiment). Error bars indicate the standard error. (2.59 MB TIF) [file pgen.1001216.s002.tif]

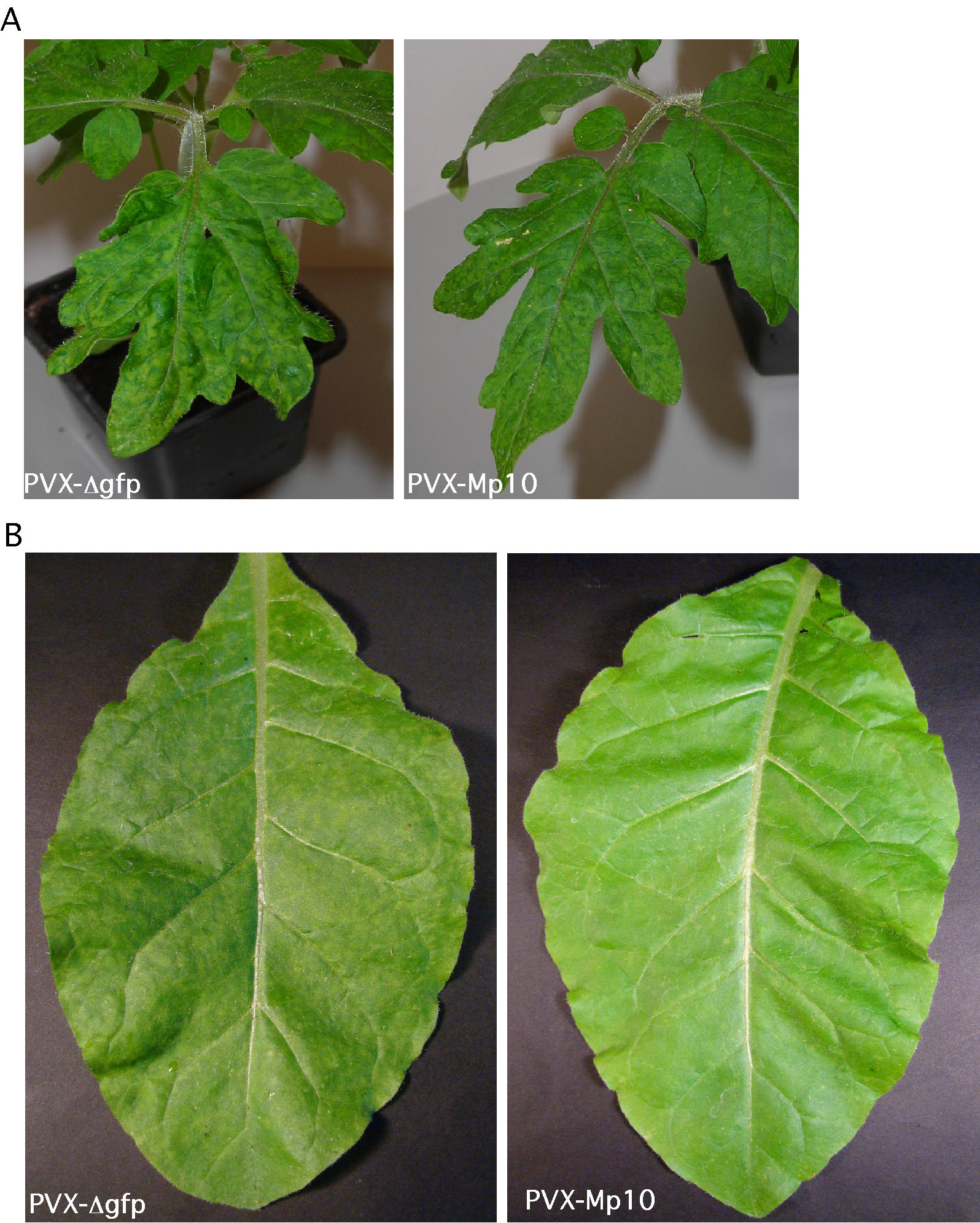

Supplement: Figure S3 — Symptoms of PVX-Mp10 infected Solanum lycopersicum (tomato) and N. tabacum plants. (A) Symptoms on a tomato plant infected with PVX-Δgfp (control) (left panel) and PVX-Mp10 (right panel). (B) Symptoms on a N. tabacum plant infected with PVX-Δgfp (control) (left panel) and PVX-Mp10 (right panel). Pictures were taken 14 days after inoculation. (8.70 MB TIF) [file pgen.1001216.s003.tif]

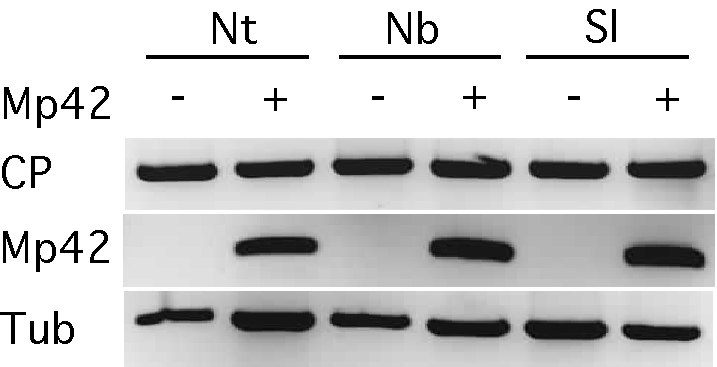

Supplement: Figure S4 — PVX-based expression of Mp42 in various plant species. Leaf tissues from N. benthamiana (Nb), N. tabacum (Nt), and Solanum lycopersicon (Sl) were collected for RNA extractions 14 days post wound-inoculation (dpwi). For semi-quantitative RT-PCR primers were used to amplify sequences corresponding to the PVX virus coat protein (CP) and Mp42. The plant tubulin gene (Tub) was used as a control for equal RNA amounts. (0.79 MB TIF) [file pgen.1001216.s004.tif]

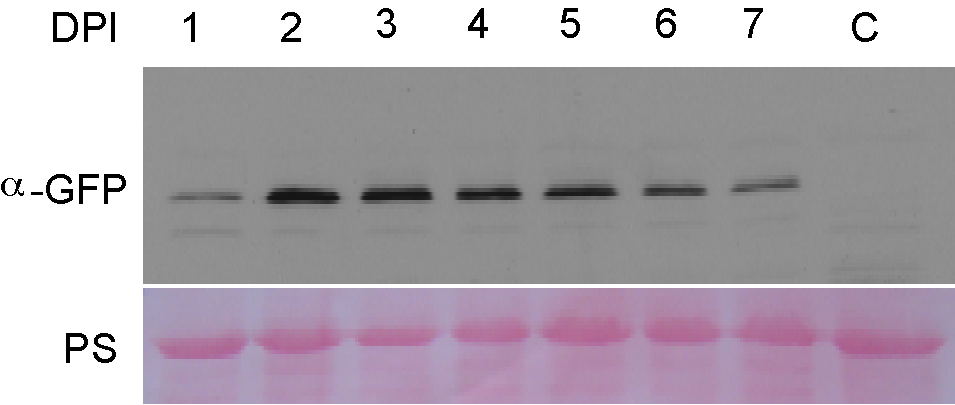

Supplement: Figure S5 — Expression of GFP in N. benthamiana leaf discs placed on water agar in a 24-well plate. Leaves were collected 24 after agroinfiltration with Agrobacterium strains expressing GFP and placed on top of water agar in a 24 wells plate. Leaf discs were collected every 24 hours from 1 to 7 days post infiltration (DPI) and ground in SDS-PAGE sample loading buffer to analyze the accumulation of GFP by western blotting with a GFP antibody. As a negative control (C) a 1-day old non-infiltrated N. benthamiana leaf disc was used. Ponceau S staining (PS) showed equal loading. (0.26 MB TIF) [file pgen.1001216.s005.tif]

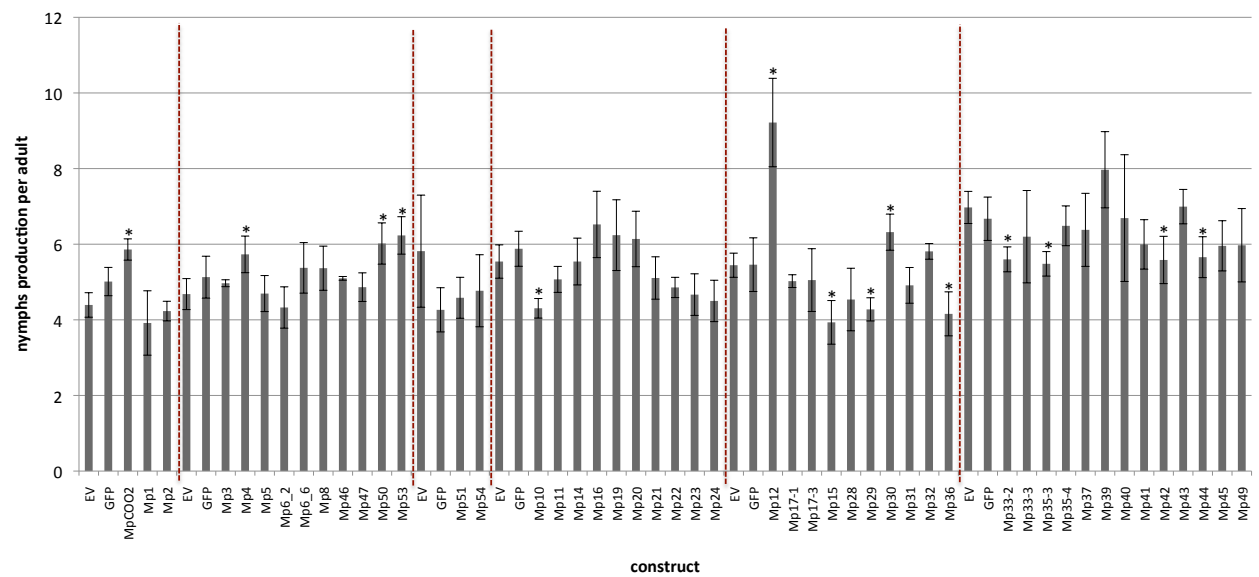

Supplement: Figure S6 — Overexpression of M. persicae candidate effector in N. benthamiana alters aphid reproductive performance (fecundity). Using the leaf disc-based assay, a set of 48 candidate effectors was expressed in N. benthamiana by agroinfiltration to screen for effects on aphid fecundity. Red dotted lines mark sets of candidates that were screened in parallel experiments. EV indicates the vector control and GFP indicates the GFP control. Nymph production was counted over a 17-day period. The average number of nymphs produced per adult was based on 3 replicated experiments. Error bars indicate the standard error. Asterisks indicate Mp candidates that were further tested in confirmation assays. (0.05 MB PDF) [file pgen.1001216.s006.pdf]

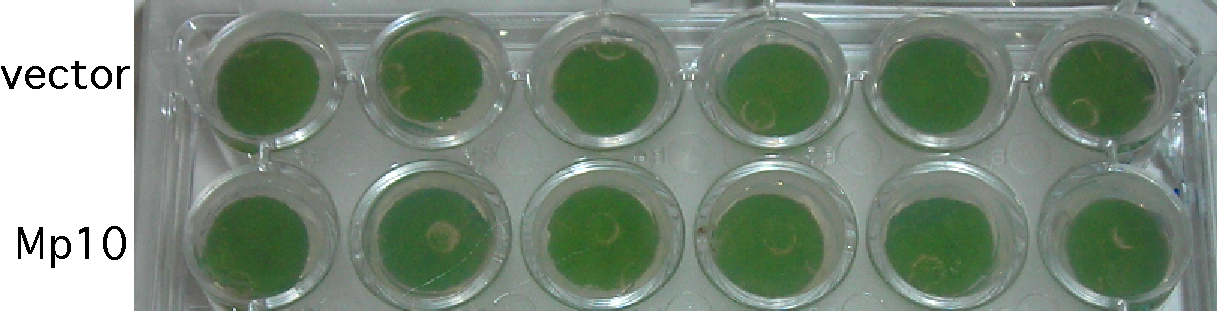

Supplement: Figure S7 — Symptoms of N. benthamiana infiltration sites expressing Mp10 during the leaf disc 24-well plate assay. Photo was taken 5 days after infiltration. (0.84 MB TIF) [file pgen.1001216.s007.tif]
