# Supplementary material for: A Functional Genomics Approach Identifies Candidate Effectors from the Aphid Species Myzus persicae (Green Peach Aphid)
Source: PLoS Genet. 2010 Nov 18;6(11):e1001216. doi: 10.1371/journal.pgen.1001216 (PMC2987835; doi:10.1371/journal.pgen.1001216)
Supplement: Table S3 — Primer table. (0.11 MB DOC) [file pgen.1001216.s010.doc]

Supplemental Table 3. Primers used in this study.

| Primername | Sequence 5’-3’ |
| --- | --- |
| MpC002-35S-F | GGAGGATCCATGAAGGTTCAGACTTCCG |
| MpC002-35S-F | GGAACTAGT TTATTAAAAATGTCTAAAGAAACG |
| Mp1-35S-F | GGAGGATCCatgGAAATGATCCAGCCGTCC |
| Mp1-35S-R | GGAACTAGTTTACAATATAGCACGGTCG |
| Mp2-35S-F | GGAGGATCCatgGAGGTTACTGAGCTCGATC |
| Mp2-35S-R | GGAACTAGTTTATTTATTTTTTGCAGCTGG |
| Mp3-35S-F | GGAGGATCCATGAAATATGGCTGGTCCACTGTGAAT |
| Mp3-35S-R | GGAACTAGTATTTCATTGCAGGCGAATGGAATTT |
| Mp4-35S-F | GGAGGATCCATGAAAATTTCTCTAATATCTCTTGG |
| Mp4-35S-R | GGAACTAGTGTCACCTTCCACACCACCAACC |
| Mp5-35S-F | GGAGGATCCATGCACGAATCGTTCTTCGGACAAG |
| Mp5-35S-R | GGAACTAGTAATATTGAACAGAATTTTATAGC |
| Mp6-35S-F | GGAGGATCCATGACACCTGTGCCAGCAGAAGACCAG |
| Mp6-35S-R | GGAACTAGTTAAATATGTGATGCTTAAATTGGCGC |
| Mp7-35S-F | GGAGGATCCATGGAAGATAACCCAGAAGAATGTGAG |
| Mp7-35S-R | GGAACTAGTCGCTACATATAATATGGATTTTAA |
| Mp8-35S-F | GGAGGATCCATGGATGTTATTGTCCGAGCTACCGCG |
| Mp8-35S-R | GGAACTAGTTTACTTGTTGTTGTCAATGCAA |
| Mp10-35S-F | GGAGGATCCATGGCGCCGCAAAAAGATGCTGTG |
| Mp10-35S-R | GGAACTAGTATTGGTAAGATTATAGGTACTTG |
| Mp11-35S-F | GGAGGATCCATGATCGGCGGATGTCCGGAATTCG |
| Mp11-35S-R | GGAACTAGTCTGCAGACGTATAGTAGTGCGTA |
| Mp12-35S-F2 | GGAAGATCTATGGCTCCTTCATCGAAATTACCT |
| Mp12-35S-R | GGAACTAGTATACAACGTACAAGCTATATCTA |
| Mp14-35S-F | GGAGGATCCATGCAAATTTTGCCATCAACTGTAGAT |
| Mp14-35S-R | GGAACTAGTTGGAACCTGAGATGACTGAATTAG |
| Mp15-35S-F | GGAGGATCCATGGATGAATTTACTATAGAAACTA |
| Mp15-35S-R2 | GGAACTAGTATTACAATATCGAATTGCAGATTTG |
| Mp16-35S-F | GGAGGATCCATGGACGGCACAGAAAAAACTCCTAAAG |
| Mp16-35S-R | GGAACTAGTAACCATGATTATCATTCTATTGTC |
| Mp17-35S-F2 | GGAAGATCTATGCAATACTCCGCTCCAGCTTACAAG |
| Mp17-35S-R | GGAACTAGTATGAAGAGAAGTTGTTTACACA |
| Mp19-35S-F | GGAGGATCCATGGCGGAAACGCAACAACAGGGGCT |
| Mp19-35S-R | GGAACTAGTTTGCAGTCGTCAACACATCGTTTG |
| Mp20-35S-F | GGAGGATCCATGGCCAACTTGACCGCCGCCGTCA |
| Mp20-35S-R | GGAACTAGTATTTGATCAGATTATCAATGT |
| Mp21-35S-F | GGAGGATCCATGGGCGACGTCATTATTCAAAAAAGGT |
| Mp21-35S-R | GGAACTAGTAATTTCACAGAGCTGATGAATATC |
| Mp22-35S-F | GGAGGATCCATGGGTGATATGATGAGGAAATCG |
| Mp22-35S-R | GGAACTAGTGTACCAGGTGGGTGAAGACGCAT |
| Mp23-35S-F | GGAGGATCCATGGCACCACAAGGTGCCTACTACG |
| Mp23-35S-R | GGAACTAGTGTTGTTGGATTCGTTGATTTCGTC |
| Mp24-35S-F | GGAGGATCCATGGCTGCAACTGGTCCTTCATCATT |
| Mp24-35S-R | GGAACTAGTGGTTGGATTAGTTGCATGGCATT |
| Mp28-35S-F | GGATCCATGGCTCACTGTCATCACGAAGGTG |
| Mp28-35S-R | GGAACTAGTGTATTTACACAATTGTGTACTTAGC |
| Mp29-35S-F | GGAGGATCCATGATCCAATGCTACCAGTGTTCTAC |
| Mp29-35S-R | GGAACTAGTGTTTGGACGGAATTCGTTACGAG |
| Mp30-35S-F | GGAGGATCCATGCAACAATATCAACCCACAACTCC |
| Mp30-35S-R | GGAACTAGTATGATGTTTGAGCCTTGTCGGAC |
| Mp31-35S-F | GGAGGATCCATGGGTAAAAAGGTGAGCGATAAGAAATT |
| Mp31-35S-R | GGAACTAGTTTAAAGTTCATAGTTATTAATAC |
| Mp32-35S-F | GGAGGATCCATGGAGTCAGACAACGAAGTCGATAC |
| Mp32-35S-R | GGAACTAGTCAAATTTTAAAGTACAATTACAA |
| Mp33-35S-F | GGAGGATCCATGGGCACAAAACTTCTTCCTGATA |
| Mp33-35S-R | GGAACTAGTGTACATGAAATTAATAAGTCTTATG |
| Mp35-35S-F | GGAGGATCCATGAGAAACGTGCATCAAACGACGAC |
| Mp35-35S-R | GGAACTAGTATTTATACAGCTAAGTAATACCATT |
| Mp36-35S-F | GGAGGATCCATGGATTCGATGTTTCAAAAACCATTG |
| Mp36-35S-R | GGAACTAGTATAAATAGTTTACAGATTTTATT |
| Mp37-35S-F | GGAGGATCCATGTTGCGCCATGATTCAGAATACAG |
| Mp37-35S-R | GGAACTAGTTATATTTTAAAGTACACAACACA |
| Mp38-35S-F | GGAGGATCCATGGAACTATTGCTAAACCTGCTG |
| Mp38-35S-R | GGAACTAGTATAATAACTAGAAATTCGATTAT |
| Mp39-35S-F | GGAGGATCCATGATTAGTATCAATCCGTTTAAAG |
| Mp39-35S-R | GGAACTAGTTTAACGAGTGTTGCCACTCTTGCAG |
| Mp40-35S-F2 | GGAAGATCTATGTCGGATAAGTTCTTTCAAACCGG |
| Mp40-35S-R | GGAACTAGTTTTAGATCAATTATTAAATGTAT |
| Mp41-35S-F | GGAGGATCCATGCAAAAACAAGAACCATCAGGAAAATGTAG |
| Mp41-35S-R | GGAACTAGTATAGTTGGTTATGTACAGTGGTGG |
| Mp42-35S-F | GGAGGATCCATGAATACAGTTAAAAAAGGTGAAGT |
| Mp42-35S-R | GGAACTAGTTTATTAACTCAATTACAATACTA |
| Mp43-35S-F | GGAGGATCCATGCAATATGCACCAGCACCGCCGGG |
| Mp43-35S-R | GGAACTAGTTTATGCCGATTGTTTCTGAGTAC |
| Mp44-35S-F | GGAGGATCCATGGAAGAAGCCCCAAAAGCCGAAG |
| Mp44-35S-R | GGAACTAGTAAAGATCACATATTTATTGTT |
| Mp45-35S-F | GGAGGATCCATGCAAGTTATGTGCAGTCAAGACAAT |
| Mp45-35S-R | GGAACTAGTATAACCACTCATCGGTGGTAGGAAT |
| Mp46-35S-F | GGAGGATCCATGCACAAATTAATAAAAGTCGATC |
| Mp46-35S-R | GGAACTAGTTAATTTTGATTTAATTTTGAGATG |
| Mp47-35S-F | GGAGGATCCATGGCTCCTGCTGAAACAATAATTGG |
| Mp47-35S-R | GGAACTAGTAATTTCTAAGTATGACGTAATGC |
| Mp49-35S-F | GGAGGATCCATGGCAATACCCATTAATTGTCCATC |
| Mp49-35S-R | GGAACTAGTTTAATCGCATCTTTTTGCTCCTC |
| Mp50-35S-F | GGAGGATCCATGAAGTCTGACAGTGAAATTGATTTG |
| Mp50-35S-R | GGAACTAGTGTGGCTAGTACTAATAATGATG |
| Mp51-35S-F | GGAGGATCCATGAATGAAATTAACGTCAAACAACTG |
| Mp51-35S-R | GGATCTAGATATAATATTGTTGAGAAATCTCCC |
| Mp53-35S-F | GGAGGATCCATGGATGTGAGTCAACAACAACAAGG |
| Mp53-35S-R | GGAACTAGTGAAGTGTGATCGGGGAGATGTCGC |
| Mp54-35S-F | GGAGGATCCATGGGAAAAGTGCCATCTTCAGATTT |
| Mp54-35S-R | GGAACTAGTTAAACTGTGATTCTGCATGGCCA |
| GFP-35S-F | GGAGGATCCATGGTGAGCAAGGGCGAGGAG |
| GFP-35S-R | GGAACTAGTTTACGTTTCTCGTTCAGCTTTTTTGTA |
| Mp10-pvx-F | GGAATCGATGGATTCACGAATTGCAGTAGTC |
| Mp10-pvx-R | GGAGCGGCCGCTTAAAATTTGACAACACCTTTTTTC |
| Mp42-pvx-F | GGAATCGATGAATACAGTTAAAAAAGGTG |
| Mp42-pvx-R | GGAGCGGCCGCTTAAACTCCACCAGATTCTGATG |
| PVX-CP-F | TAGCACAACACAGCCCATAGG |
| PVX-CP-R | GGCAGCATTCATTTCAGCTTC |
| Tub-F | ATCGCATCCGAAAGCTTGCAG |
| Tub-R | ACATCAACATTCAGAGCTCCATC |
| MpActin-F | CGGTTCAAAAACCCAAACCAG |
| MpActin-R | TGGTGATGATTCCCGTGTTC |
